# Navigating Nutritional Challenges on the Way to Maximum Athletic Performance

**DOI:** 10.3390/nu16244385

**Published:** 2024-12-20

**Authors:** Giannis Arnaoutis

**Affiliations:** Department of Nutrition and Dietetics, School of Health Science and Education, Harokopio University, El. Venizelou Ave. 70, 17671 Athens, Greece; garn@hua.gr

## Introduction

Nutritional interventions play a vital role in the amelioration of athletic performance, with the use of specific, safe, and ergogenic supplements, such as creatine, caffeine, sodium bicarbonate, beta-alanine, and beetroot juice, demonstrating their capacity to enhance several crucial aspects of sports performance such as strength, endurance, and recovery [1]. In the present Special Issue, a thorough, full review by Jose Alonso and his colleagues addresses the efficacy and safety of these sport supplements. Moreover, many other newly studied supplements offer further potential, showing promise in specific contexts, such as increasing stamina, enhancing focus, or accelerating recovery, but their integration into various sports disciplines requires careful evaluation. Nevertheless, key questions remain regarding their precise dosing and mechanisms, as well as their long-term effects on health and performance. Therefore, this Special Issue has focused on the growing body of evidence on supplementation strategies and innovative ergogenic techniques, including studies that have explored their efficacy across disciplines, emphasizing personalized approaches in order to maximize benefits.

The published papers also underscore a critical need: the importance of empowering the athletes with accurate sports nutrition knowledge and access to trained sports nutritionists. The data consistently document that athletes exhibit a serious lack of sports nutrition knowledge, provoking them to exhibit inadequate dietary intake, putting them consequently at risk of inappropriate dietary choices that could ultimately undermine their athletic performance and/or increase the risk of injury [2]. It has also to be highlighted that this risk is evident in multiple sports, as well as during different training periods or even during competition. This relationship between athletes and nutrition professionals can bridge the well-documented gap between science and practice, ensuring at the same time the safe and effective implementation of supplementation protocols.

Finally, future research should prioritize integrating ergogenic supplementation into periodized training regimens that align with the specific demands of elite athletic populations, for both men and women. Such integration would allow for better synchronization between supplementation, training cycles, and recovery phases. In addition to refining these protocols, emphasis must be placed on the long-term monitoring of the impacts of supplementation to identify potential risks, interactions, and diminishing returns with chronic use. Furthermore, nutrition education for athletes, coaches, and training personnel is crucial. Accessible, evidence-based resources empower individuals to make informed decisions about supplementation, reducing at the same time the reliance on anecdotal advice or unregulated products. To conclude, this balanced approach seems to be the key that will ultimately help athletes achieve peak performance while safeguarding their health in their respective sports.
